# Serum calcium as a biomarker of clinical severity and prognosis in patients with coronavirus disease 2019

**DOI:** 10.18632/aging.103526

**Published:** 2020-06-25

**Authors:** Jia-Kui Sun, Wen-Hao Zhang, Lei Zou, Ying Liu, Jing-Jing Li, Xiao-Hua Kan, Lian Dai, Qian-Kun Shi, Shou-Tao Yuan, Wen-Kui Yu, Hong-Yang Xu, Wei Gu, Jian-Wei Qi

**Affiliations:** 1 Department of Intensive Care Unit, Nanjing First Hospital, Nanjing Medical University, Nanjing, Jiangsu Province, China; 2 Department of Intensive Care Unit, Lishui People's Hospital, Nanjing, Jiangsu Province, China; 3 Department of Intensive Care Unit, Drum Tower Hospital, Nanjing University, Nanjing, Jiangsu Province, China; 4 Department of Intensive Care Unit, Wuxi People's Hospital, Nanjing Medical University, Wuxi, Jiangsu Province, China; 5 Department of Isolation Units, Tongji Hospital, Huazhong University of Science and Technology, Wuhan, Jiangsu Province, China

**Keywords:** hypocalcemia, vitamin D, parathyroid hormone, organ injury, prognosis, COVID-19

## Abstract

The aim of this study was to investigate the correlations between serum calcium and clinical outcomes in patients with coronavirus disease 2019 (COVID-19). In this retrospective study, serum calcium levels, hormone levels and clinical laboratory parameters on admission were recorded. The clinical outcome variables were also recorded. From February 10 to February 28, 2020, 241 patients were enrolled. Of these patients, 180 (74.7%) had hypocalcemia on admission. The median serum calcium levels were 2.12 (IQR, 2.04-2.20) mmol/L, median parathyroid hormone (PTH) levels were 55.27 (IQR, 42.73-73.15) pg/mL, and median 25-hydroxy-vitamin D (VD) levels were 10.20 (IQR, 8.20-12.65) ng/mL. The serum calcium levels were significantly positively correlated with VD levels (*P* =0.004) but negatively correlated with PTH levels (*P* =0.048). Patients with lower serum calcium levels (especially ≤2.0 mmol/L) had worse clinical parameters, higher incidences of organ injury and septic shock, and higher 28-day mortality. The areas under the receiver operating characteristic curves of multiple organ dysfunction syndrome, septic shock, and 28-day mortality were 0.923 (*P* <0.001), 0.905 (*P* =0.001), and 0.929 (*P* <0.001), respectively. In conclusion, serum calcium was associated with the clinical severity and prognosis of patients with COVID-19. Hypocalcemia may be associated with imbalanced VD and PTH levels.

## INTRODUCTION

In December 2019, clusters of acute pneumonia cases of unclear etiology were identified in Wuhan City, the capital of Hubei Province in China [1–3]. The pathogen has been reported as a novel coronavirus named severe acute respiratory syndrome coronavirus 2 (SARS-CoV-2). The World Health Organization (WHO) has made the assessment that coronavirus disease 2019 (COVID-19) can be characterized as a pandemic because the disease is still spreading rapidly around the world, especially in the United States, Spain, and Russia [4]. As of May 26, a total of 82993 cases (4634 deaths) were confirmed in China, including 50340 cases (3869 deaths) in Wuhan city [5].

The National Health Commission of China has issued a series of diagnosis and treatment recommendations and suggested classifying the disease into four grades: mild, moderate, severe and critical [5]. Recent studies have reported the clinical characteristics and prognosis of the varied severity grades of COVID-19 [1, 2, 6–8]. The underlying mechanisms of the novel coronavirus leading to disease exacerbation and organ dysfunction remain to be further explored. Due to the high mortality and the lack of effective treatments in critically ill patients [7, 9], early identification and prediction of these patients are crucial. What are the risk factors for severe illness or death [10]? How can we identify groups that are most likely to have poor outcomes so that we can focus prevention and treatment efforts [10]? These studies are needed. Huang et al [8] reported that patients admitted to the intensive care unit (ICU) had more severe clinical symptoms and more abnormal serum parameters. However, fewer studies have been published that confirm an early and sensitive biomarker to estimate the disease severity and prognosis of COVID-19. During our clinical work against the COVID-19 epidemic in Wuhan, we observed a high incidence of hypocalcemia in critically ill patients. Therefore, we hypothesized that serum calcium levels were associated with the disease severity and prognosis of patients with COVID-19. This study was performed to test this hypothesis and explore the causes of hypocalcemia.

## RESULTS

A total of 241 patients with confirmed COVID-19 were enrolled in this clinical retrospective study. The median age was 65 (IQR, 55-72) years, and 129 (53.5%) were women. Of the patients, 192 (79.7%) were classified as severe (167/214, 69.3%) or critical (25/214, 10.5%). Fever (108/214, 44.8%) and cough (65/214, 27.0%) were the main initial symptoms. One hundred and eighty (180/214, 74.7%) patients had hypocalcemia on admission. The detailed clinical data of the patients are presented in Table 1. A total of 231 patients were discharged from the hospital, and the median hospital stay was 25 (IQR, 17-32) days. MODS developed in 17 (7.1%) patients, and septic shock developed in 6 (2.5%) patients. Ten (10/241, 4.1%) patients died within 28 days of admission, and all of the decedents were critically ill. In other words, the 28-day mortality of critically ill patients was 40.0% (10/25).

**Table 1:** Demographic data and clinical parameters.

**Variables**	
***Categorical variables***	***N (%)***
Sex (Male: Female)	112:129
Initial symptoms or signs	
Fever	108 (44.8%)
Cough	65 (27.0%)
Chest tightness or pain	22 (9.1%)
Fatigue	10 (4.1%)
Dyspnea	9 (3.7%)
Diarrhea	7 (2.9%)
Pharyngalgia	5 (2.1%)
Myalgia	5 (2.1%)
Nausea or vomiting	4 (1.7%)
Abdominal pain	3 (1.2%)
Other	3 (1.2%)
Classifications	
Mild	0 (0%)
Moderate	49 (20.3%)
Severe	167 (69.3%)
Critical	25 (10.4%)
Organs injury	
ARDS	19 (7.9%)
Liver injury	16 (6.6%)
AKI	14 (5.8%)
Cardiac injury	12 (5.0%)
Septic shock	6 (2.5%)
Need for NIV /HFNC	7 (2.9%)
Need for MV	12 (5.0%)
Need for CRRT	7 (2.9%)
Discharged	94 (39.0%)
Death	10 (4.1%)
***Continuous variables***	***Median (IQR)***
Age (years)	65 (55-72)
Days from onset to admission	13 (10-16)
Blood parameters	
Calcium (mmol/L)	2.12 (2.04-2.20)
CRP (mg/L)	6.30 (1.70-34.85)
WBC (10^9^/L)	5.48 (4.55-7.15)
Lymphocyte (10^9^/L)	1.26 (0.93-1.63)
ALT (U/L)	22.0 (14.0-36.0)
Albumin (g/L)	35.6 (31.6-38.8)
Creatinine (umol/L)	66.0 (56.0-80.0)
TNI (pg/mL)	3.80 (1.95-7.45)
D-dimer (ug/mL)	0.73 (0.34-1.42)
Worst SpO2 (%)	97.0 (96.0-98.0)

ARDS, acute respiratory distress syndrome; AKI, acute kidney injury; NIV, noninvasive ventilation; HFNC, high-flow nasal cannula; MV, mechanical ventilation; CRRT, continuous renal replacement therapy; IQR: interquartile ranges; CRP, C-reactive protein; WBC, white blood cells; ALT, alanine aminotransferase; TNI, troponin I; SpO2, pulse oxygen saturation.

### Serum calcium and clinical variables

The median serum calcium levels were 2.12 (IQR, 2.04-2.20) mmol/L on admission. We divided the patients into three groups based on the serum calcium values: ≤2.0 mmol/L (defined as group A, *n* = 43), 2.0-2.2 mmol/L (defined as group B, *n* = 137), and >2.2 mmol/L (defined as group C, *n* = 61). As shown in Table 2, significant differences in the clinical variables except for serum creatinine were found among the three groups, and the same differences were found between groups A and B (*P* <0.05). There were also differences in the clinical variables except for WBC count (*P* =0.07) and serum creatinine (*P* =0.244) between groups A and C, whereas differences in the variables except for WBC count (*P* =0.60), ALT (*P* =0.839), the lowest SpO2 (*P* =0.328), and serum creatinine (*P* =0.635) were found between groups B and C. These results indicated that patients with lower serum calcium levels had worse clinical variables.

**Table 2:** Serum calcium and clinical parameters.

	**Group A (*n*= 43)**	**Group B (*n*= 137)**	**Group C (*n*= 61)**	***P* value**
Calcium (mmol/L)	1.96 (1.91-2.00)	2.11 (2.06-2.13)	2.22 (2.21-2.26)	<0.001
CRP (mg/L)	47.4 (20.5-105.7)	6.3 (1.9-25.8)	2.0 (0.8-6.2)	<0.001
WBC (10^9^/L)	6.58 (4.57-9.01)	5.36 (4.38-6.79)	5.29 (4.67-6.96)	0.042
Lymphocyte (10^9^/L)	0.75 (0.50-1.19)	1.27 (1.01-1.63)	1.53 (1.17-1.75)	<0.001
ALT (U/L)	32.0 (18.0-51.0)	20.0 (14.0-34.5)	20.0 (13.0-35.5)	0.027
Albumin (g/L)	30.6 (28.2-32.6)	35.6 (31.7-38.4)	40.0 (36.1-43.2)	<0.001
Creatinine (umol/L)	66.0 (61.0-79.0)	67.0 (55.5-80.0)	64.0 (57.0-80.0)	0.565
TNI (pg/mL)	8.80 (3.90-16.70)	3.40 (1.95-6.20)	2.50 (1.90-4.55)	<0.001
D-dimer (ug/mL)	1.30 (0.74-8.29)	0.68 (0.34-1.37)	0.43 (0.27-0.80)	<0.001
Worst SpO2 (%)	96.0 (90.0-97.0)	97.0 (96.0-98.0)	97.0 (96.0-98.0)	<0.001

Group A, the serum calcium values: ≤2.0 mmol/L; Group B, the serum calcium values: 2.0-2.2 mmol/L; Group C, the serum calcium values: >2.2 mmol/L; CRP, C-reactive protein; WBC, white blood cells; ALT, alanine aminotransferase; TNI, troponin I; SpO2, pulse oxygen saturation.

Of the 241 patients, 26 were tested to determine levels of parathyroid hormone (PTH) and 25-hydroxy-vitamin D (VD) according to clinical needs. The median serum calcium level of the 26 patients was 2.13 (IQR, 2.03-2.16) mmol/L. The median PTH level was 55.27 (IQR, 42.73-73.15) pg/mL, and the median VD level was 10.20 (IQR, 8.20-12.65) ng/mL. All of these patients had low levels of VD (VD deficiency).

**Figure 1:**
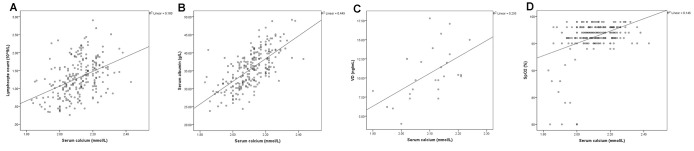
The serum calcium levels were positively correlated with lymphocyte count (**A**, *P* <0.001), and albumin (**B**, *P* <0.001), 25-hydroxy-vitamin D (VD) (**C**, *P* =0.004), and lowest SpO2 (**D**, *P*< 0.001) levels.

**Figure 2:**
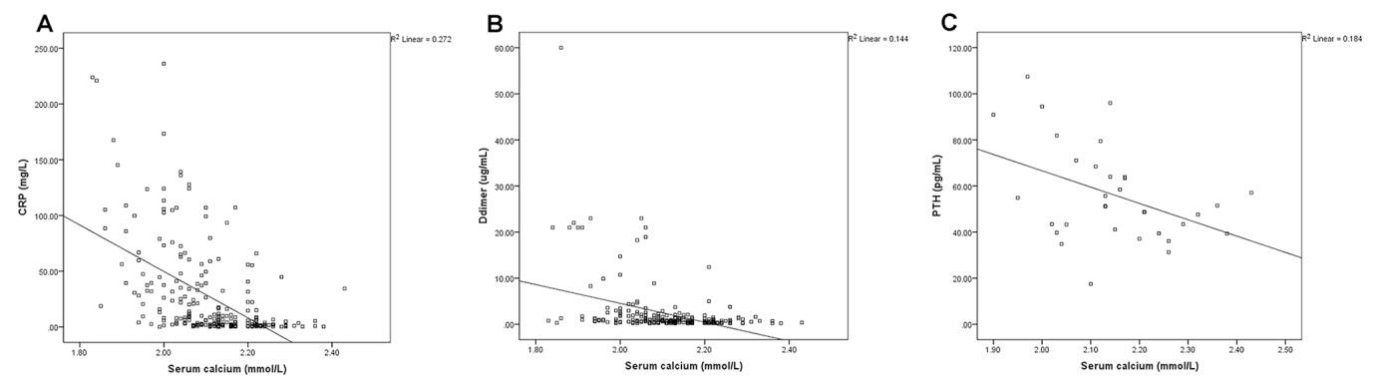
The serum calcium levels were negatively correlated with C-reactive protein (CRP) (**A**, *P*< 0.001), D-dimer (**B**, *P*< 0.001) and parathyroid hormone (PTH) (**C**, *P* =0.048) levels.

The SPSS scatterplots and correlation analyses of serum calcium and the blood biomarkers are shown in Figures 1 and 2. The serum calcium levels were significantly positively correlated with lymphocyte count (Figure 1A, *P* <0.001), albumin levels (Figure 1B, *P* <0.001), VD levels (Figure 1C, *P* =0.004), and lowest SpO2 (Figure 1D, *P*< 0.001), whereas they are significantly negatively correlated with CRP (Figure 2A, *P*< 0.001), D-dimer (Figure 2B, *P*< 0.001) and PTH (Figure 2C, *P* =0.048) levels. These results indicated that hypocalcemia may be associated with imbalanced VD and PTH in the acute phase of COVID-19.

### Serum calcium and clinical severity

ARDS developed in 19 of 241 (7.9%) patients, and liver injury developed in 16 (6.6%), AKI developed in 14 (5.8%), and cardiac injury developed in 12 (5.0%) patients during the research period. Twelve patients received MV, and 7 patients received CRRT. As shown in Table 3, significant differences in the clinical severity and outcome variables were found among the abovementioned three groups (A, B, and C) (*P* <0.001) and between groups A and C (P <0.001). There were also differences in all these variables except for liver injury incidence (*P* =0.201). No differences were found between groups B and C (*P* >0.05). These results indicated that patients with serum calcium values ≤2.0 mmol/L had higher 28-day mortality, and a higher incidence of organ injury. Moreover, the serum calcium values were significantly lower in patients who died and in patients with MODS, septic shock, and organ injury, requiring MV or CRRT (*P* <0.001) (Table 4).

**Table 3:** Serum calcium and clinical variables of severity and outcomes.

	**Group A (*n*= 43)**	**Group B (*n*= 137)**	**Group C (*n*= 61)**	***P* value**
Death	10 (23.3%)	0 (0.0%)	0 (0.0%)	<0.001
MODS	14 (32.6%)	3 (2.2%)	0 (0.0%)	<0.001
Septic shock	6 (14.0%)	0 (0.0%)	0 (0.0%)	<0.001
ARDS	15 (34.9%)	3 (2.2%)	1 (1.6%)	<0.001
Liver injury	6 (14.0%)	9 (6.6%)	1 (1.6%)	<0.001
AKI	8 (18.6%)	5 (3.6%)	1 (1.6%)	<0.001
Cardiac injury	7 (16.3%)	5 (3.6%)	0 (0.0%)	<0.001
Need for MV	12 (27.9%)	0 (0.0%)	0 (0.0%)	<0.001
Need for CRRT	7 (16.3%)	0 (0.0%)	0 (0.0%)	<0.001

Group A, the serum calcium values: ≤2.0 mmol/L; Group B, the serum calcium values: 2.0-2.2 mmol/L; Group C, the serum calcium values: >2.2 mmol/L; MODS, multiple organ dysfunction syndrome; ARDS, acute respiratory distress syndrome; AKI, acute kidney injury; MV, mechanical ventilation; CRRT, continuous renal replacement therapy.

**Table 4:** Values of serum calcium in relation to the presence or absence of clinical variables of severity and outcomes.

**Clinical variables**	**Presence**			**Absence**		***P* value**
	**serum calcium**	***n***		**serum calcium**	***n***	
Death	1.96 (1.90-2.00)	10		2.12(2.05-2.20)	231	< 0.001
MODS	1.95 (1.88-2.00)	17		2.13 (2.06-2.20)	224	< 0.001
Septic shock	1.98(1.92-2.00)	6		2.12(2.05-2.20)	235	< 0.001
ARDS	1.95 (1.90-2.00)	19		2.13(2.06-2.20)	222	< 0.001
Liver injury	2.04 (1.96-2.06)	16		2.12(2.05-2.20)	225	< 0.001
AKI	1.97(1.87-2.07)	14		2.12(2.05-2.20)	227	< 0.001
Cardiac injury	2.00 (1.90-2.07)	12		2.12(2.05-2.20)	229	< 0.001
Need for MV	1.94(1.89-2.00)	12		2.12(2.05-2.20)	229	< 0.001
Need for CRRT	1.91(1.84-1.94)	7		2.12(2.05-2.20)	234	< 0.001

MODS, multiple organ dysfunction syndrome; ARDS, acute respiratory distress syndrome; AKI, acute kidney injury; MV, mechanical ventilation; CRRT, continuous renal replacement therapy.

**Figure 3:**
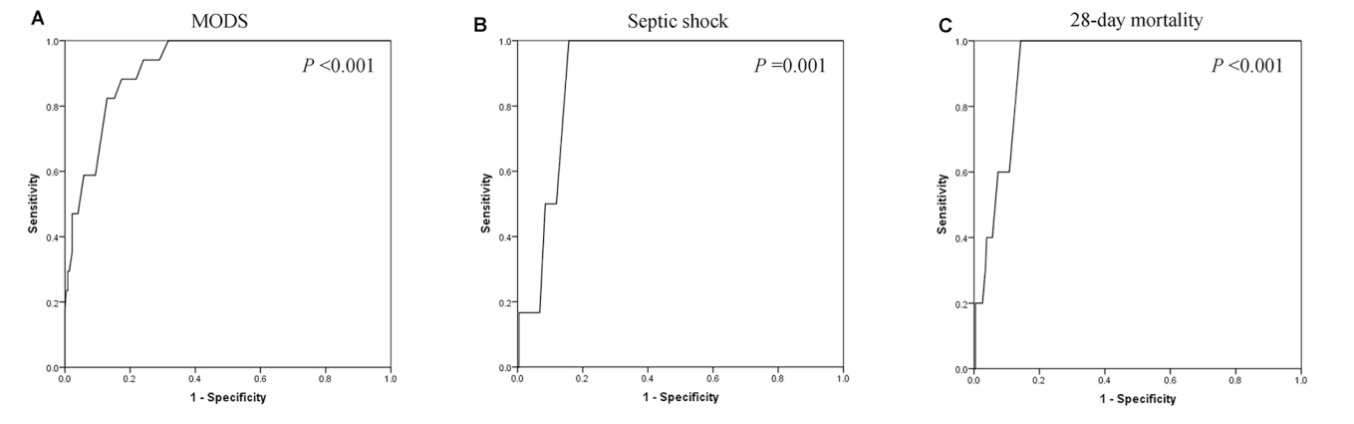
The areas under the receiver operating characteristic curves of multiple organ dysfunction syndrome (MODS) (**A**), septic shock (**B**), and 28-day mortality (**C**) were 0.923 (*P* <0.001), 0.905 (*P* =0.001), and 0.929 (*P* <0.001), respectively.

**Figure 4:**
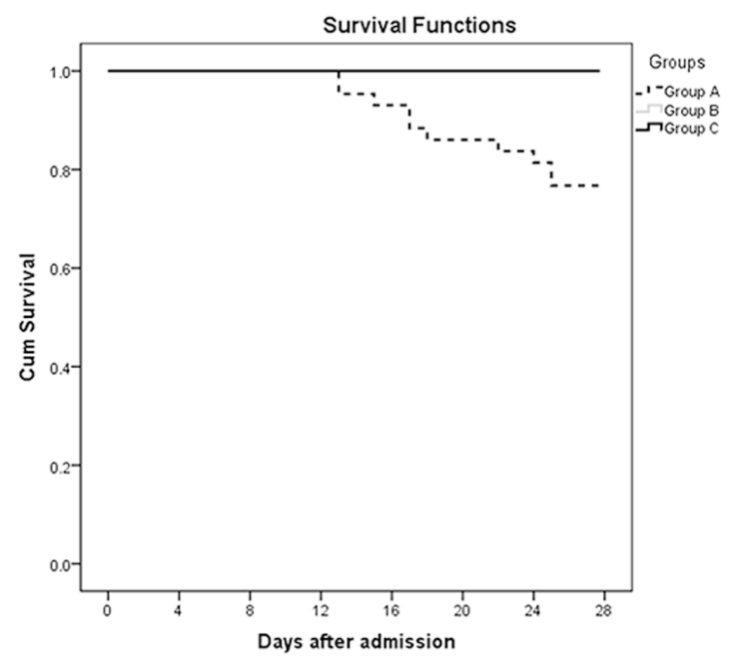
The survival curves at 28 days after admission of the three groups: Group A (serum calcium values ≤2.0 mmol/L, *n* =43), Group B (serum calcium values 2.0-2.2 mmol/L, *n* =137), and Group C (serum calcium values >2.2 mmol/L, *n* =61) (*P* <0.001).

ROC curves were also performed to assess the associations between serum calcium and MODS, septic shock, and 28-day mortality. As shown in Figure 3, the area under the curves (AUCs) of MODS (Figure 3A), septic shock (Figure 3B), and 28-day mortality (Figure 3C) were 0.923 (*P* <0.001), 0.905 (*P* =0.001), and 0.929 (*P* <0.001), respectively. Optimal cut-off points of serum calcium values were derived from the ROC curves. The optimal cut-off point for MODS was 2.035 mmol/L, the sensitivity was 88.2%, and the specificity was 82.6%. The optimal cut-off point for septic shock was 2.01 mmol/L, the sensitivity was 100.0%, and the specificity was 84.3%. The optimal cut-off point for 28-day mortality was 2.01 mmol/L, the sensitivity was 100.0%, and the specificity was 85.7%. Figure 4 shows the significant differences in the 28-day mortality among groups A, B, and C. The 28-day mortality of group A was significantly higher than that of groups B or C (*P* <0.001). No difference was found between groups B and C (*P* >0.05).

## DISCUSSION

This clinical retrospective study investigated the correlations between serum calcium and clinical severity and outcomes in patients with COVID-19. The incidence of hypocalcemia was 74.7%. We found that patients with lower serum calcium levels (especially ≤2.0 mmol/L) had worse clinical variables, higher incidences of MODS and septic shock, and higher 28-day mortality. Hypocalcemia may be associated with imbalanced VD and PTH in the acute phase of COVID-19. The overall mortality was 4.1% (10/241), whereas the mortality of critically ill patients was increased to 40.0% (10/25).

The WHO declared that COVID-19 was a pandemic because the disease is still spreading rapidly around the world [4]. More than 5700000 patients have been diagnosed with COVID-19 worldwide, and nearly 360000 have died [4]. Although the National Health Commission of China and the WHO have issued a series of diagnosis and treatment recommendations, the mortality of critically ill patients is still extremely high. The underlying mechanisms of the novel coronavirus causing organ dysfunction are yet unknown. It is crucial to identify the risk factors for severe illness or death [10]. However, few reports have been published to establish an early and sensitive biomarker to predict the disease severity and prognosis of COVID-19. In this study, we found that serum calcium levels were associated with the disease severity and prognosis of patients with COVID-19.

Hypocalcemia is common in critically ill patients. The causes of hypocalcemia include oversecretion of PTH, VD deficiency, decreased dietary intake, hypoproteinemia, hypomagnesemia drug interactions, and so on [15]. Hypocalcemia was defined as a serum calcium level less than 2.2 mmol/L in our clinical laboratory. At present, there is no specific severity grading system for hypocalcemia. Previous studies reported that serum calcium levels less than approximately 2.0 mmol/L were associated with worse clinical outcomes in critically ill patients [15–17]. Therefore, we divided patients into three groups based on serum calcium values of ≤2.0 mmol/L, 2.0-2.2 mmol/L, and >2.2 mmol/L. We found that patients with serum calcium values ≤2.0 mmol/L had higher 28-day mortality, and a higher incidence of organ injury. The findings of this study were consistent with previous reports. However, this was the first study to investigate the correlations between serum calcium and clinical outcomes in patients with COVID-19. These results suggested that correcting hypocalcemia could be an important strategy to improve the prognosis of patients with COVID-19, especially for patients with serum calcium values less than 2 mmol/L. In addition, our study also revealed that hypocalcemia was associated with hypoproteinemia and imbalanced VD and PTH in the acute phase of COVID-19. Hypoproteinemia and VD deficiency were also common and correlated with increased mortality in critically ill patients [18, 19]. Therefore, improvement of hypoproteinemia and imbalanced hormone levels may also be useful in the treatment of COVID-19.

Increased CRP, ALT, TNI, and D-dimer levels and lymphocytopenia were present in most critical COVID-19 patients [2, 7, 8]. Our results also showed that patients with lower serum calcium values had higher levels of CRP, ALT, TNI, and D-dimer and lower lymphocyte counts. The serum calcium values were significantly correlated with lymphocyte count and CRP and D-dimer levels. Moreover, CRP and D-dimer were also indicators to predict the prognosis of critically ill patients [19]. The findings of this study were consistent with previous reports and confirmed that serum calcium levels were associated with the disease severity and prognosis of patients with COVID-19. The lung is the main organ affected by this disease. In this study, the median lowest SpO2 was 97.0% (IQR, 96.0%-98.0%; range, 80%-99%), and the SpO2 values were significantly positively correlated with serum calcium levels. Patients with serum calcium values ≤2.0 mmol/L had higher ARDS incidence, while patients with ARDS also had lower serum calcium values. These phenomena indicated that hypocalcemia might be crucial in the development of ARDS. Early diagnosis and treatment of hypocalcemia may alleviate organ injury in the acute phase of COVID-19.

Some limitations of the study should be discussed. Because of our single-center retrospective design and small sample size, the results might be inconclusive, and the accuracy should be confirmed by large-scale prospective clinical studies. Moreover, because the study was not based on pathophysiological models, the results were hypothesis generating, and the exact mechanisms of hypocalcemia and VD deficiency should be tested by more fundamental experiments. In addition, the values of serum calcium were of total calcium rather than ionized calcium in this study, which may not precisely reflect the extent of decreased calcium.

## CONCLUSIONS

In conclusion, this retrospective clinical study found that the incidence of hypocalcemia and VD deficiency was very high in patients with COVID-19. Hypocalcemia may be associated with imbalanced VD and PTH levels. Patients with lower serum calcium levels (especially ≤2.0 mmol/L) had worse clinical variables, higher incidences of MODS and septic shock, and higher 28-day mortality. The overall mortality of COVID-19 was 4.1%, whereas the mortality of critically ill patients was 40.0%.

## MATERIALS AND METHODS

### Patients

From February 10 to February 28, 2020, adult patients (age ≥18 years) with confirmed COVID-19 admitted to our specialized isolation units, Tongji Hospital of Huazhong University of Science and Technology in Wuhan, were enrolled in this clinical retrospective study. Patients with chronic organ dysfunction (e.g., hepatic or renal dysfunction), terminal cancer, immunodeficiency, and a history of long-term use of hormones were excluded. Written informed consent was waived by our institutional review board because this was a retrospective study that assessed deidentified data and included no potential risk to patients. The diagnosis of COVID-19 was made according to the WHO interim guidance and the recommendations of the National Health Commission of China [4, 5], and confirmed by RNA detection of SARS-CoV-2 in the clinical laboratory of Tongji Hospital.

### Definitions

An identified case of COVID-19 was defined as a positive finding by real-time reverse transcriptase–polymerase chain reaction (RT-PCR) assay of nasal and pharyngeal swab specimens [4, 5, 7]. Only laboratory-confirmed cases were enrolled in the analysis. The clinical classifications of COVID-19 were in accordance with the Chinese recommendations [5]: Mild, with minor clinical symptoms (e.g., fever, cough) without imaging manifestations. Moderate, with fever or respiratory tract infection symptoms with imaging indicating pneumonia. Severe, met any of the following, I—respiratory distress and respiratory rate ≥30 breaths/min; II—pulse oxygen saturation (SpO2) ≤93% at rest; or III—arterial partial pressure of oxygen (PaO2)/ fraction of inspired oxygen (FiO2) ≤300 mmHg (1 mmHg =0.133 kPa). Critical, met any of the following, I—respiratory failure with mechanical ventilation (MV); II—shock; or III—multiple organ failure requiring ICU treatment. Hypocalcemia was defined as a serum calcium level less than 2.2 mmol/L in our clinical laboratory. Sepsis was defined as life-threatening organ dysfunction caused by a dysregulated host response to infection, and septic shock was defined as a subset of sepsis with circulatory and cellular/metabolic dysfunction that is associated with a higher risk of mortality [11]. The diagnostic criteria of acute respiratory distress syndrome (ARDS) were in accordance with the Berlin definitions [12]. The definitions of acute kidney injury (AKI) were based on the 2012 Kidney Disease: Improving Global Outcomes (KDIGO) guidelines [13]. Cardiac injury was defined if serum levels of cardiac biomarkers (e.g., troponin I) were more than twice the reference upper limit or new abnormalities were found in electrocardiography and echocardiography [2]. Liver injury was defined if serum levels of hepatic biomarkers (e.g., alanine aminotransferase) were more than twice the reference upper limit or if there was disproportionate elevation of alanine aminotransferase (ALT) and aspartate aminotransferase (AST) levels compared with alkaline phosphatase levels [14]. Multiple organ dysfunction syndrome (MODS) was defined as the combined dysfunction of two or more organs.

### Data collection

The baseline clinical characteristics, including age, sex, days from onset to admission, initial symptoms or signs, and clinical classifications were collected from electronic medical records, and all laboratory tests were performed according to the clinical needs of patients. The levels of serum calcium, C-reactive protein (CRP), ALT, albumin, creatinine, troponin I (TNI), and plasma D-dimer and white blood cell (WBC) count, lymphocyte count, and the lowest SpO2 within 24 hours of admission were recorded. The hormone levels associated with blood calcium (e.g., parathyroid hormone, 25-hydroxy-vitamin D) were also recorded. All blood parameters were detected by the clinical laboratory of Tongji Hospital. Moreover, the numbers of patients with ARDS, AKI, cardiac injury, liver injury, septic shock and MODS and patients receiving noninvasive ventilation (NIV), high-flow nasal cannula (HFNC), MV, and continuous renal replacement therapy (CRRT) were also recorded. The primary endpoints were the development of septic shock, MODS, and 28-day mortality. The secondary endpoints were the other disease severity parameters (e.g., organ injury or not).

### Statistical analysis

The Kolmogorov-Smirnov test was first performed to test the normal distribution of the data. Normally distributed data were expressed as the means ± standard deviation and were compared by t tests. Abnormally distributed data were expressed as the medians (interquartile ranges, IQR) and were compared by the Mann-Whitney U test or the Kruskal-Wallis test. Categorical variables were presented as absolute numbers or percentages and were analyzed using the χ^2^ test or Fisher’s exact test. To take into account the repeated nature of the variables, analysis of variance (ANOVA) for repeated measurements of the general linear model was implemented. Receiver operating characteristic (ROC) curves were used to evaluate the associations between serum calcium and septic shock, MODS, and 28-day mortality. IBM Statistical Package for the Social Sciences (SPSS, version 22.0, NY, USA) software was used for statistical analysis, and *P* <0.05 was considered statistically significant. SPSS scatterplots and a correlation analysis were performed to evaluate the relevance between serum calcium and blood biomarkers. The statistical methods of this study were reviewed by Qiao Liu, a biostatistician from the Center for Disease Control and Prevention of Jiangsu Province in China.
